# Effects of Sand Type and Alkali Equivalent on Drying Shrinkage and Microstructure of Seawater–Sea Sand Alkali-Activated Slag Concrete

**DOI:** 10.3390/ma18051005

**Published:** 2025-02-25

**Authors:** Jianbin Zhang, Sixiang Kang, Yanran Shen, Chenhao Song, Haoliang Lei, Wei Xie, Xianyun Chen, Jize Wang, Wenda Wu, Xuefang Wang

**Affiliations:** 1 Xiamen Municipal Engineering Design Institute Co., Ltd., Xiamen 361000, China; zjb348@jsti.com (J.Z.); lhl327@jsti.com (H.L.); xw335@jsti.com (W.X.); cxy392@jsti.com (X.C.); 2 College of Advanced Manufacturing, Fuzhou University, Quanzhou 362200, China; kangsixing66@gmail.com (S.K.); m15170669103@163.com (J.W.); 3 College of Civil Engineering, Fuzhou University, Fuzhou 350108, China; yanran61997@163.com; 4 College of Civil Engineering, Putian University, Putian 351100, China; a2539311197@gmail.com

**Keywords:** alkali-activated concretes, seawater, sea sand, alkali equivalent, drying shrinkage

## Abstract

The use of seawater and sea sand for the preparation of alkali-activated concretes plays an important role in reducing carbon emissions and alleviating resource scarcity. However, both alkali-activated concretes and products made from seawater and sea sand tend to exhibit significant drying shrinkage. Therefore, this study investigates the effect of the addition of seawater and sea sand on the basic properties and drying shrinkage properties of alkali-activated concretes, and regulates the properties of seawater and sea sand alkali-activated slag (SSAS) concretes with the key parameter of the alkali equivalent. Finally, the mechanism of SSAS drying shrinkage improvement was investigated using XRD, TG, MIP, and SEM. The results show that the addition of seawater and untreated sea sand accelerates the hydration reaction of alkali-excited cement and can significantly reduce its drying shrinkage. A moderate increase in alkali content can improve the compressive strength and reduce the drying shrinkage. However, an excessively high alkali content reduces the flexural strength. Finally, we propose the existence of a quantitative relationship between drying shrinkage, mass loss, and the evaporable water content, which better explains the mechanism of shrinkage variation. These effects are mainly attributed to changes in microstructure and phase composition. This study provides theoretical support for engineering applications of seawater–sea sand alkali-activated materials.

## 1. Introduction

Cement, as one of the most widely used engineering materials in the world, contributes to roughly 8% of global carbon emissions annually due to its production, significantly impacting the sustainability goals of the construction sector [1]. Carbon sequestration is one of the methods used to reduce carbon emissions in the cement industry. From 1930 to 2013, carbonated cement materials cumulatively sequestered 4.5 GtC, offsetting 43% of the CO_2_ emissions from cement production during the same period [2]. Another widely explored approach is the search for low-carbon alternative materials. Alkali-activated concrete has emerged as a promising substitute, utilizing industrial by-products such as blast furnace slag and fly ash instead of cement clinker. The appropriate selection of the raw materials, activators, and mix design can significantly reduce carbon emissions in the cement production process [3,4]. At the same time, this approach also enhances the mechanical strength [5] and durability [6], attributes mainly credited to the formation of C-A-S-H gel during the alkali activation process.

Additionally, the production of cement concrete consumes a vast amount of fresh water and river sand, not only placing pressure on the environment but also being restricted due to the increasingly scarce resources. More than 2 billion tons of fresh water are consumed annually for concrete production [7]. Research by the World Meteorological Organization predicts that by 2025, more than half of the world’s population will face challenges with insufficient drinking water [8]. Furthermore, the excessive extraction of river sand damages river ecosystems [9] and induces natural disasters [10,11]. Given that 71% of the Earth’s surface is covered by seawater, and that sea sand resources are almost inexhaustible, using seawater and sea sand to produce concrete has become an inevitable choice to address resource shortages and promote the development of sustainable building materials. Particularly for island and coastal regions, where resources are constrained, the adoption of seawater and sea sand in concrete production offers a practical solution to reduce costs and expedite construction timelines.

In regions rich in marine resources, there has been a shift towards using sea sand in the preparation of cement and concrete, accompanied by the development of advanced desalination technologies [12,13]. XRD and petrographic analyses indicate that the mineral composition of sea sand closely mirrors that of river sand, which is commonly used in concrete production [14]. Moreover, research suggests that concrete produced with sea sand may possess superior mechanical properties [15,16,17,18]. Pan [19] and others found that incorporating sea sand and seawater in cement enhances the formation of ettringite phases, which in turn refines the cement’s microstructure. This refinement results in a more compact material with reduced porosity, thereby bolstering its mechanical strengths. However, sea sand that has not undergone desalination contains a large amount of shells, which could adversely affect the concrete structure [8,20,21]. More seriously, the high chloride ion content in both seawater and sea sand poses a significant risk. These ions can dissolve in the concrete’s pore water and migrate towards steel reinforcements, exacerbating corrosion risks and compromising structural safety [22]. Furthermore, the presence of sulfates in seawater can cause chemical attacks on cement hydration products, potentially leading to expansion and cracking [23]. The magnesium ions in seawater may also react with cement hydration products, forming brucite and M-S-H gel, which can decrease the concrete’s strength [18]. These challenges have traditionally limited the broader adoption of seawater and sea sand in concrete applications. Such concerns significantly limit the broader adoption of seawater and sea sand in concrete applications.

However, alkali-activated materials show promising potential in addressing these challenges. The C-A-S-H gel formed during the hydration process of alkali-activated concrete demonstrates strong chloride-binding capacity [24,25,26], potentially mitigating the corrosion risks. Additionally, research has shown that alkali-activated slag concrete exhibits enhanced resistance to sulfate attack compared to ordinary Portland cement concrete [27]. Research focusing on alkali-activated slag in marine settings has revealed that such materials, even after extended exposure to seawater, tend to exhibit increased strength over time [28]. Alkali-activated slag concrete demonstrates enhanced resistance to chloride ion erosion in comparison to OPC, attributable to its denser microstructure, which seawater finds more challenging to penetrate [29]. This suggests a superior resilience of the alkali-activated slag system to marine conditions over the silicate system, positioning alkali-activated slag as an optimal choice for environments subjected to harsh marine influences. There is a growing body of research on alkali-activated materials prepared with seawater and sea sand. Investigations by Li [30] into the thermal behavior of seawater–sea sand alkali-activated concrete and slag paste at elevated temperatures, in comparison to traditional Portland cement, concluded that the degradation of concrete is independent of the water and sand types utilized. Further studies have shown that alkali-activated slag mortar blended with seawater maintains higher compressive strength after undergoing wet–dry cycles than its freshwater and cement–slag counterparts [31]. These insights underscore the importance of further research into how different types of water and sand, along with their mixing ratios, influence the performance of seawater–sea sand alkali-activated slag concrete, encouraging a deeper examination of these materials’ potential.

Drying shrinkage is a critical factor in the design and construction phases, particularly in marine environments where shrinkage-induced cracks can hasten the infiltration of corrosive ions, elevating the risk of corrosion in steel reinforcements. Alkali-activated concrete, characterized by its fine pore structure and robust capillary action, is prone to higher drying shrinkage levels, with previous research indicating that its shrinkage range is 2 to 4 times higher than that of traditional concrete [32,33]. The use of seawater and sea sand impacts the shrinkage behavior of OPC as well [34,35], yet comprehensive studies on the shrinkage performance of SSAS remain scarce. It has been observed that alkali-activated slag cement formulated with seawater and sea sand tends to exhibit increased drying shrinkage [36,37]. For SSAS, factors such as the type of water and sand, the activator’s nature and quantity, and the mix composition influence its drying shrinkage [34]. Yang [33] compared the drying shrinkage performance between SSAS and conventional alkali-activated concrete; however, the variations and underlying mechanisms of drying shrinkage under different conditions remain undefined. Thus, delving into the drying shrinkage mechanisms of seawater–sea alkali-activated slag materials is crucial for understanding their potential in engineering applications.

This study aimed to assess the impacts of seawater and sea sand on the drying shrinkage of SSAS, compared to using fresh water and river sand. Additionally, it sought to understand how drying shrinkage in SSAS can be regulated by adjusting the alkali equivalent. Advanced microscopic techniques such as XRD, TG, SEM, and MIP were used to investigate the effects on the hydration products, microstructure, and pore distribution. This research seeks to understand the mechanisms behind drying shrinkage, providing a theoretical foundation for using SSAS in marine engineering and promoting sustainable development through resource efficiency. Of particular interest is the potential application in non-reinforced concrete products, especially concrete blocks, where steel corrosion is not a concern. The absence of steel reinforcement in concrete blocks eliminates the primary obstacle to using seawater and sea sand in concrete—the risk of chloride-induced corrosion. Therefore, concrete blocks represent an ideal application for SSAS technology, offering a practical solution to reduce freshwater consumption and river sand mining in coastal regions. Understanding the shrinkage behavior and its control mechanisms is crucial for optimizing SSAS mixtures for such applications, thereby expanding the practical implementation of this environmentally friendly construction material.

## 2. Materials and Methods

### 2.1. Materials

#### 2.1.1. Sand

The desalinated sea sand (DS) was collected from Fujian Province, China, and was obtained through washing and drying treatments. The undesalinated sea sand (US) was also sourced from the same location but was left untreated. The river sand (RS) was taken from a sand factory in Longgang District, Shenzhen City, and was in its natural state. The physical indicators of the three types of sand were determined and the results are shown in Table 1. The desalinated sea sand was obtained through the following steps. First, the sea sand was washed with tap water to remove surface salt and impurities (the washing continued until the wash water became clear). The pre-washed sand was soaked in water with a water-to-sand ratio of 3:1 and thoroughly stirred for 10 min. The wash water was then drained, and this process was repeated three times. Due to shells being lighter and prone to damage during stirring, the shell content of the sea sand decreased after the desalination process. The shell content of the sand was determined using the hydrochloric acid dissolution method specified in JGJ 52-2006 [38]. The specific steps were as follows. The representative sample was oven-dried at 105 ± 5 °C to a constant mass (m_1_). The sample was placed in a beaker, and a hydrochloric acid solution was gradually added with continuous stirring until no further gas evolution was observed. Additional acid was added to ensure a complete reaction. The sample was washed 5 times with distilled water, carefully avoiding sand particle loss. The washed sample was dried at 105 ± 5 °C to reach a constant mass (m_2_). The shell content (W_b_) was calculated using the following equation:

w_b_ = [(m_1_ − m_2_)/m_1_ × 100%] − w_c_

w_b_—Shell content (%)

m_1_—Initial sample mass (g)

m_2_—Sample mass after acid treatment (g)

w_c_—Mud content (%)

After calculation, the fineness modulus of the DS was found to be 1.84, classifying it as zone III fine sand. The fineness modulus of the US and RS were 2.83 and 3.03, classifying them as zone II medium sand. The chemical compositions of the sands are shown in Table 2.

#### 2.1.2. Water

All water used in the experiments was deionized water produced by a laboratory pure water machine. Artificial seawater was used in this study instead of natural seawater to ensure experimental reproducibility and eliminate variability in the composition. Natural seawater’s composition can vary significantly with location, season, and depth, potentially affecting the reliability and comparability of the results. The artificial seawater was prepared according to ASTM D1141-89 standard [39] by mixing analytical-grade NaCl, MgCl_2_, Na_2_SO_4_, CaCl_2_, KCl, and NaHCO_3_, with the detailed mix proportions presented in Table 3.

#### 2.1.3. Slag

**Figure 1:**
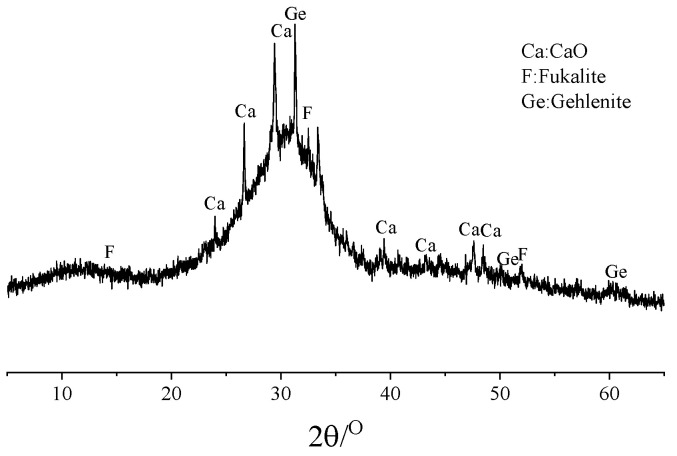
XRD image of slag powder.

The slag was sourced from Hebei Province, China, and was categorized as S95-grade granulated blast furnace slag. Its basic physical properties are listed in Table 4. The slag sample was ground and passed through a 75 μm sieve, then pressed into a powder using a hydraulic press for an XRF analysis, with the results shown in Table 5. An XRD analysis was also conducted on the slag (ground to below 45 μm), with the results depicted in Figure 1. The XRD analysis indicates the presence of noticeable peaks for CaO, fukalite, and gehlenite in the slag powder.

#### 2.1.4. Activators and Retarders

The alkali activator was anhydrous Na_2_SiO_3_ from Tianjin Zhiyuan Co., Ltd. (Tianjin, China). The retarding agent was 99.5% pure borax from Tianjin Dengfeng Chemical Reagent Factory (Tianjin, China).

### 2.2. Preparation Method

#### 2.2.1. Cement Preparation Methods

The preparation of the alkali-activated slag mortar involved a mechanochemical method. Initially, dried slag and the alkali activator were combined in a specified ratio. This mixture was then transferred into a ball milling jar (WZM-15 × 2), containing a mix of steel balls. These balls, in diameters of 30 mm, 20 mm, and 10 mm, were used in an equal ratio of 1:1:1, amounting to a total weight of 15 kg. The proportion of the combined mass of the slag and alkali activator to the steel balls was 1:7.5, indicating that the mixture weighed 2 kg. The jar was sealed and placed in a ball mill, where it underwent high-speed milling at 85 revolutions per minute for one hour. Following the milling process, the resultant powder was sifted through a sieve, yielding the alkali-activated slag cement required for the experiment.

#### 2.2.2. Preparation Methods for SSAS Mortar

The preparation of the SSAS mortar involved mixing the slag cement with sea sand at a mass ratio of 1:2. Seawater was then added according to the predetermined water–binder ratio. The specific proportions of the mortar are shown in Table 6.

### 2.3. Testing and Analysis

#### 2.3.1. Basic Performance Test

The flowability of the mortar was assessed using a flow table test, following the GB/T 2419-2005 standard [40].

The specimens were prepared according to GB/T 17671-1999 [41]. These specimens were cured at 20 ± 2 °C with over 95% humidity. Their compressive and flexural strengths were measured at 3, 7, and 28 days using a DYE-300 tester. Six specimens were tested for compressive strength and three for flexural strength, with outliers exceeding ±10% excluded to ensure accurate averages.

#### 2.3.2. Drying Shrinkage and Mass Loss Test

**Figure 2:**
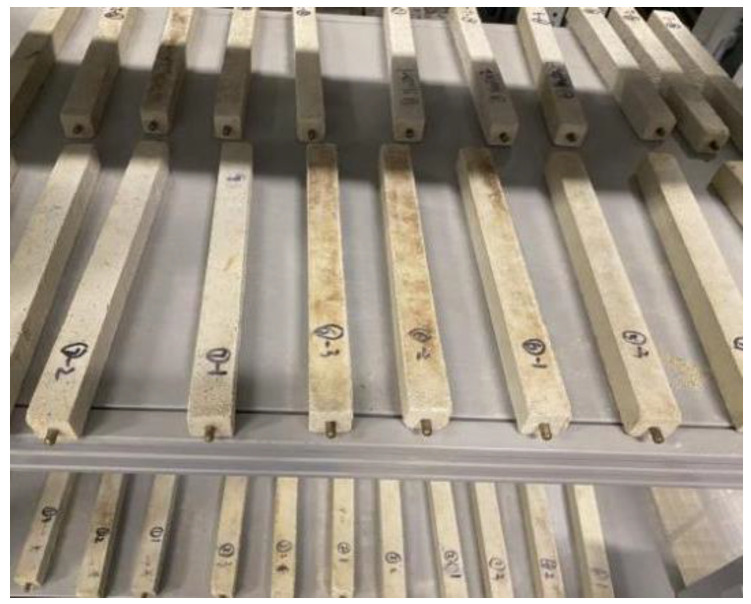
Drying and shrinking samples.

The test method followed the JGJ/T 70-2009 [42] and JC/T 603-2004 standards [43], using a 25 mm × 25 mm × 280 mm shrinkage steel mold with copper pins at both ends (The samples are shown in Figure 2). The mortar was cast in two layers, tamped 24 times, smoothed, and covered with cling film. Demolding occurred 48 ± 2 h after water addition. The specimen was then water-cured at 20 ± 2 °C for 24 h, followed by drying in a chamber at 20 ± 3 °C and 50 ± 4% humidity for 4 h. The initial length (*L*_0_) and mass (*M*_0_) were recorded on day 0, and subsequent measurements were taken at 3, 7, 14, 28, 42, 56, 90, and 180 days to calculate the drying shrinkage (*S*_t_) and mass loss (*M*_l_).

(1) $$S_{t}=\frac{L_{0}-L_{t}}{250}\times 100$$

(2) $$M_{l}=\frac{M_{0}-M_{t}}{M_{0}}\times 100$$

#### 2.3.3. XRD Test

Layered powder samples were obtained from concrete specimens using a profile grinding machine, then sieved through an 80 μm sieve. The powder was soaked in isopropanol to stop hydration, with three replacements every 24 h. After 7 days, the samples were dried in a vacuum oven. The mineral composition of the dried slag mortar was analyzed using an X-ray diffractometer (Rigaku Miniflex 600, Akishima, Japan), scanning from 5° to 80° at a step size of 0.02° and a speed of 10°/min.

#### 2.3.4. TG Test

The instrument raised the temperature to 1000 °C at a heating rate of 10 K/min under room temperature conditions, followed by isothermal conditions for 20 min. The TG curve of the sample was automatically recorded during this process, and a first-order derivative of the temperature yielded the DTG curve.

#### 2.3.5. MIP Test

A concrete cutting machine was used to cut the specimen into 5 mm thin slices. The core area of each slice was then crushed into 2–3 g of sample for testing. The samples underwent a hydration halting treatment as described in Section 2.3.4. After this process, they were dried in a vacuum oven for 7 days and stored indoors to prevent surface cracks from excessive drying. The pore structure was analyzed using an AutoPore 9500 pressure porosimeter (MicroActive, Austin, TX, USA), with a pressure range of 0.10 to 61,000.00 psia, to measure the porosity, pore size distribution, density, and other properties.

The International Union of Pure and Applied Chemistry (IUPAC) classifies pores into micropores (<1.25 nm), mesopores (1.25–25 nm), macropores (25–5000 nm), and microcracks (>5000 nm) [44]. In this study, due to the absence of micropores, the pore sizes were categorized following the IUPAC standards into mesopores, macropores, and microcracks.

#### 2.3.6. SEM Test

SEM testing was performed using a Hitachi Regulus 8100 scanning electron microscope (Chiyoda, Japan). Cross-sections of dried and gold-sputtered mortar samples were scanned to analyze their micro-morphology and elemental distribution. The SEM samples were prepared following the MIP sampling method, with the samples chosen to have diameters and thicknesses not exceeding 5 mm.

## 3. Results and Discussion

### 3.1. Flowability

#### 3.1.1. Impact of Seawater and Sea Sand

**Figure 3:**
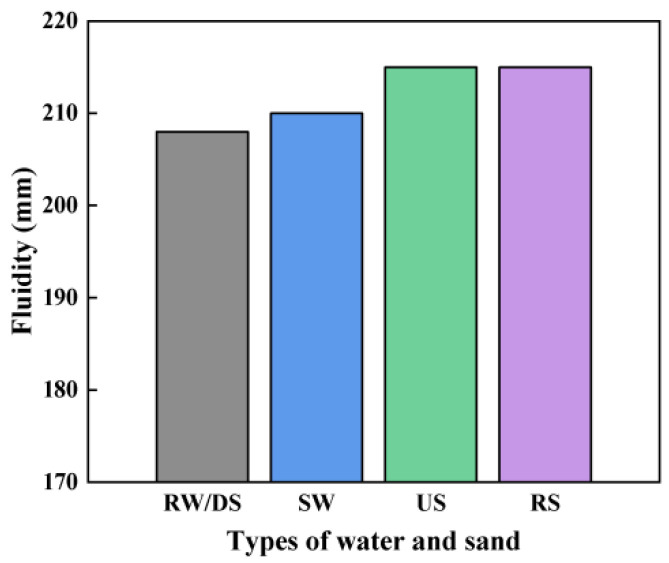
Effects of water and sand type on SSAS flowability.

Figure 3 shows the impacts of different water and sand types on the SSAS sample flowability. The mortar flowability rates when prepared with both SW and RW exhibited similar results. The groups using US and RS had nearly identical flowability, while the DS group showed slightly lower flowability.

This aligns with the existing literature [36,45], which suggests that the finer particle size and higher specific surface area of sea sand in the DS group likely increased the water demand, reducing the workability [46]. Contrary to some studies, the presence of shells in the sea sand did not significantly affect the flowability in the US samples, indicating that the impurities had minimal impact. This finding emphasizes the importance of considering both the physical and chemical properties of materials in construction [47].

#### 3.1.2. Impact of Alkali Equivalent

**Figure 4:**
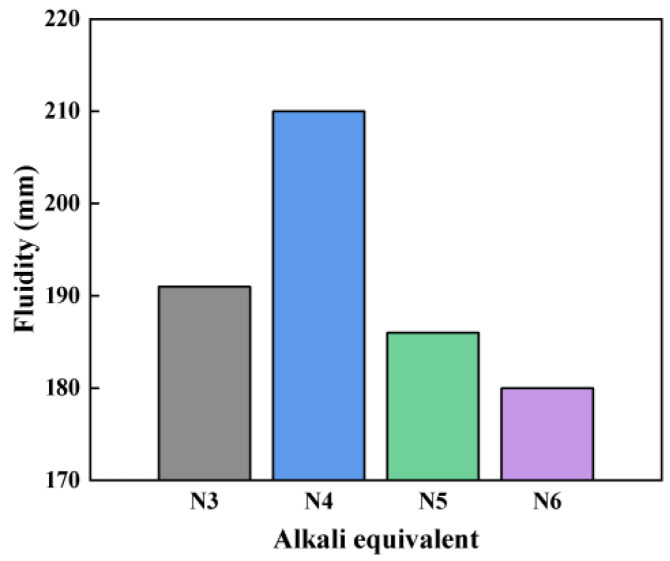
Effect of alkali equivalent on SSAS flowability.

Figure 4 illustrates that the flowability of the SSAS samples initially increases with the alkali equivalent, peaking at 4% with a maximum flowability of 210 mm, and then decreases. Despite the potential for improvement with a retarder, the flowability for N5 and N6 decreases by 11.42% and 14.28%, respectively, compared to N4. Lower alkali equivalents result in less particle dispersion, diminishing the flowability. As the alkali equivalent rises from 3% to 4%, the particle dispersion and flowability improve. However, increasing the alkali equivalent to 6% causes an increase in polymer formation, which reduces the mixture’s fluidity [48].

### 3.2. Mechanical Properties

#### 3.2.1. Impact of Seawater and Sea Sand

**Figure 5:**
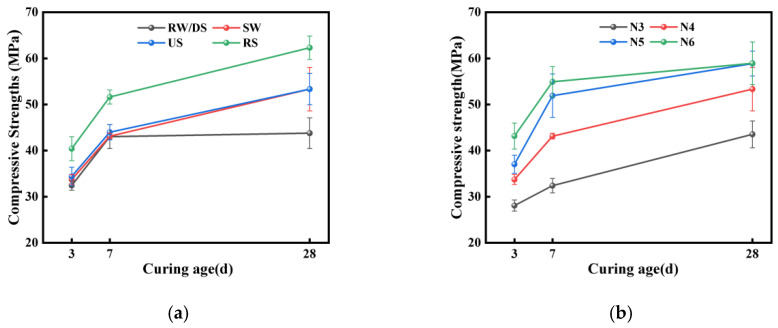
Plot of changes in compressive strength of samples: (**a**) effects of water and sand types; (**b**) effect of alkali equivalent.

Figure 5a show how the different water and sand types affected the SSAS mortar’s mechanical properties. The use of seawater enhanced the SSAS mortar’s compressive strength at 3, 7, and 28 days by 5.25%, 1.65%, and 18.44%, respectively. Although some research suggests that freshwater may yield slightly higher strengths in alkali-activated materials [49], the differences are generally minor. Most studies agree that seawater improves the mechanical properties of alkali slag materials [50] and traditional Portland cement matrices [51,52]. This enhancement is attributed to chloride ions in seawater, which form chlorides such as CaCl_2_, accelerating the early hydration and promoting C-(A)-S-H gel formation [36]. Additionally, the seawater raises the mortar’s pH, which is crucial for alkali-activated concrete’s polymerization [53].

The samples exhibited rapid early-stage strength development, with the 3-day compressive strengths reaching about 64% of their 28-day strengths. At 28 days, the US group’s compressive strength was 18.44% higher than the DS group’s, likely due to the higher chloride content in the unwashed sea sand accelerating the hydration [36]. Additionally, the mechanical performance of the specimens prepared with RS was superior to those prepared with different SS types, aligning with the findings of most scholars [54,55]. This could be attributed to the smooth surface of sea sand weakening the bonding performance between the binder and aggregate, reducing the filling effect and affecting the mechanical performance of the concrete [56]. Additionally, sea sand contains more salt, and the presence of a small amount of crushed shells in the sea sand used in this experiment may also cause some degree of damage to the mechanical properties of concrete [9].

While this enhanced strength from US is ideal for non-reinforced applications such as concrete blocks, the high chloride content limits its use in reinforced concrete. The DS group, showing lower strength but better chloride control, may be more suitable where steel reinforcement is required. This highlights the importance of sand selection based on specific application requirements.

#### 3.2.2. Impact of Alkali Equivalent

Figure 5b show how increasing the alkali equivalents from 3% to 6% enhanced the compressive strength of SSAS, with the highest value recorded at 60.9 MPa for the N6 group after 28 days. This improvement is attributed to the increased slag solubility and accelerated hydration product formation with higher alkali levels, boosting the mechanical strength [57,58]. However, the use of excessive alkali equivalents can lead to decreased compressive strength, as higher alkali levels can overly accelerate reactions and affect the structural integrity [59,60]. Rees’ research [61] suggests that there exists an optimal pH value in the alkali system, at which the polymerization reaction rate of silicates is the fastest. Therefore, differences in experimental results may arise depending on the chosen alkali equivalents.

### 3.3. Drying Shrinkage Properties

#### 3.3.1. Impact of Seawater and Sea Sand

**Figure 6:**
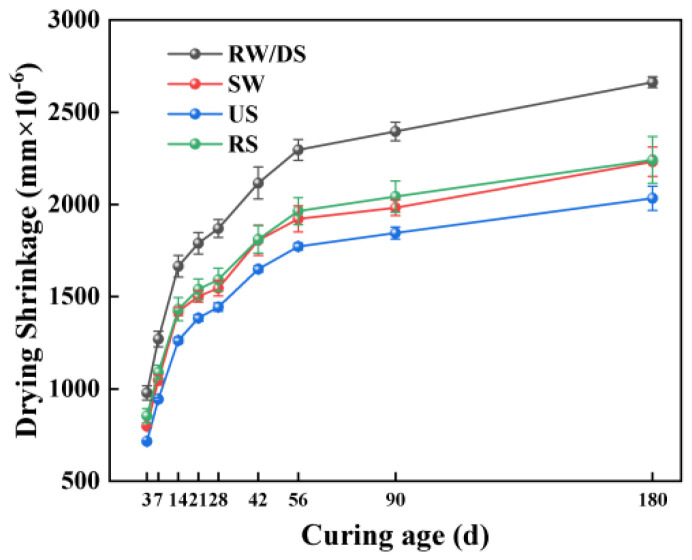
Effects of water and sand type on drying shrinkage of SSAS mortar.

Figure 6 illustrates the impacts of water and sand types on the drying shrinkage characteristics of the SSAS mortar. The results indicate that the drying shrinkage of all samples increased with the curing time, exhibiting rapid early development of drying shrinkage. Compared to RW, the SW mortars at 28, 56, and 180 days experienced reductions in drying shrinkage by 16.06%, 16.23%, and 16.28%, respectively, showing seawater’s efficacy in reducing shrinkage. This reduction is due to chloride ions in seawater accelerating hydration, filling pores, and reducing moisture loss [62]. Additionally, the hygroscopic and moisture-retaining properties of chlorides themselves also help reduce drying shrinkage [63].

The sand type impacts for drying shrinkage followed the order of DS > RS > US, with the least shrinkage found in the US group. This is also due to its higher chloride ion content, which accelerates hydration, resulting in more hydration products that fill the pores and reduce drying shrinkage [62]. Additionally, the US group’s higher CaCO_3_ content further densifies the porous structure, contributing to lower shrinkage [33]. The effectiveness of CaCO_3_ in reducing drying shrinkage has been recognized in both alkali-activated and traditional silicate systems [64,65]. Despite being prepared from sea sand, the DS group mortar exhibited the highest drying shrinkage rate. The desalinated sea sand used in this experiment had the highest density among the three types. When using the same mass of sand, the DS mortar led to an increase in shrinkage values due to having a larger volume proportion of the cement paste. Additionally, the DS group exhibited the highest shrinkage rate, possibly due to its higher density and water demands, increasing the cement paste volume and accelerating water loss, leading to greater shrinkage [46].

#### 3.3.2. Impact of Alkali Equivalent

**Figure 7:**
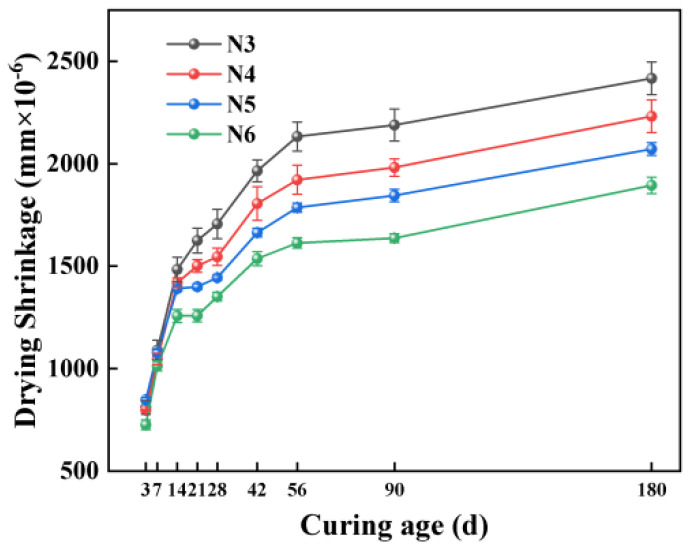
Effect of alkali equivalent on drying shrinkage of SSAS mortar.

Figure 7 illustrates that the drying shrinkage in SSAS decreases as the alkali equivalent increases, with the N6 group showing the lowest shrinkage. Specifically, the drying shrinkage in the N6 group decreased by 10.44%, 24.34%, and 21.62% at 3, 56, and 180 days, respectively, compared to the N3 group. This finding contrasts with some studies suggesting higher alkali equivalents increase drying shrinkage due to a finer pore structure and higher mesopore ratio [66,67]. The variance may stem from the different sodium silicate moduli (Na_2_O/SiO_2_) used, affecting the hydration products and microstructure.

Some research [68] supports the idea that lower alkali equivalents lead to incomplete hydration, filling pores and reducing shrinkage, while higher levels promote Ca(OH)_2_ precipitation [69] and C-A-S-H gel formation [70,71], reducing mesopores and shrinkage. Mixtures with better flowability, suggesting interconnected pores, facilitate moisture loss. A 6% alkali equivalent appears optimal for minimizing drying shrinkage in SSAS, although a further microscopic analysis is needed to confirm the mechanisms.

### 3.4. Mass Loss

#### 3.4.1. Impact of Seawater and Sea Sand

**Figure 8:**
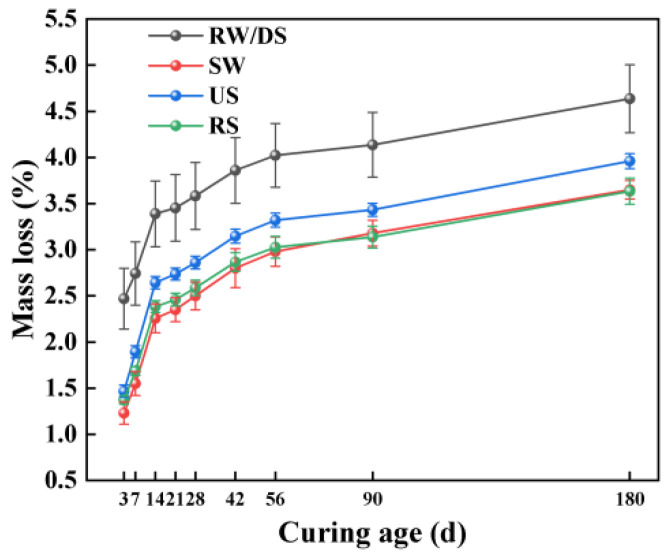
Effects of water and sand type on mass loss of SSAS mortar.

Figure 8 highlights the effects of using different water and sand types on the mass loss of SSAS under drying conditions. The data show that the mortar made with SW had a notable reduction in mass loss mortar compared to RW, with decreases of 30.16%, 25.87%, and 21.34% at 28, 56, and 180 days, respectively. This indicates seawater’s effectiveness in minimizing moisture loss in alkali-activated mortar. Despite this suppressive effect lessening over time, it remains significant in reducing the drying shrinkage rate.

In terms of sand types, the unwashed US group showed the highest mass loss across all ages tested, followed by the DS group, with RS experiencing the least mass loss. Compared to RS, DS exhibited 38.22%, 32.67%, and 27.82% more mass loss at 28, 56, and 180 days, respectively. Although the US group showed higher mass loss than RS, it exhibited lower drying shrinkage. The finer particle size of sea sand necessitates a greater water demand for the mortar, contributing to the increased mass loss [46]. Although the US group recorded higher mass loss than RS, it showed reduced drying shrinkage, suggesting that changes in sand type impact mass loss and drying shrinkage differently. Impurities, such as shells in unwashed sea sand, might add CaCO_3_, altering the mortar’s microstructure and porosity [9] and thereby reducing the drying shrinkage but increasing the mass loss due to the higher water requirements. These observations highlight the complex relationship between the sand type, mass loss, and drying shrinkage in SSAS, underscoring the importance of comprehensive material performance considerations in the formulation of alkali-activated slag mortar with seawater and sea sand.

#### 3.4.2. Impact of Alkali Equivalent

**Figure 9:**
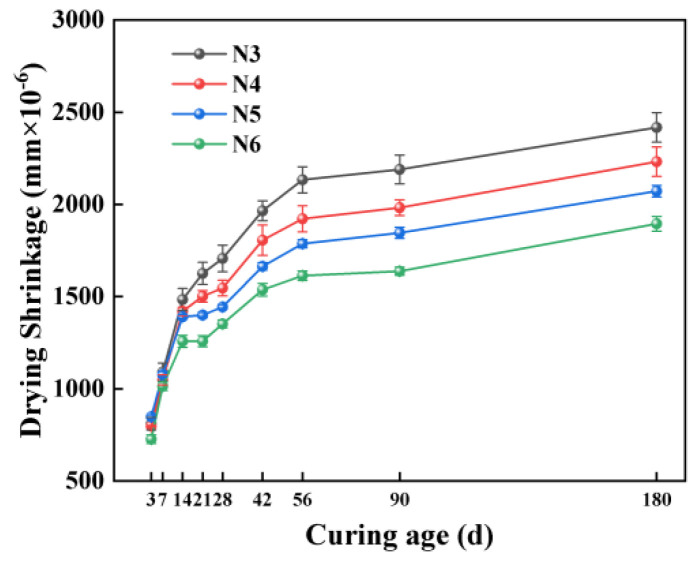
Effect of alkali equivalent on mass loss of SSAS mortar.

Figure 9 illustrates how the alkali equivalent affects the mass loss of SSAS mortar. When the alkali equivalent surpasses 5%, the impact on the mass loss becomes minimal, showcasing a slight downward trend. This observation highlights a marked sensitivity to alkali equivalent increases between 3% and 5%, where changes in mass loss are notably significant. Across the spectrum from 3% to 6% alkali equivalents, there is an overall reduction in mass loss, particularly within the initial 14 days, with the rate of loss tapering off in the later stages. Specifically, the N6 group exhibited reductions in mass loss of 48.49%, 46.89%, and 42.79% at 28, 56, and 180 days, respectively, when compared to the N3 group.

This phenomenon is consistent with previous research findings showing that as the Na_2_O content increases, the mass loss decreases [72]. This effect might be due to the increased alkali equivalent raising the pH value inside the mixture, accelerating the dissolution and reaction processes of particles such as slag, thereby reducing the content of free water in the system and consequently the overall mass loss [56]. This phenomenon indicates that by adjusting the alkali equivalent, it is possible not only to optimize the mechanical properties and drying shrinkage behavior of alkali-activated slag mortar but also to effectively control the mass loss under drying conditions, enhancing the material’s overall performance and durability.

#### 3.4.3. Relationship Between Mass Loss and Drying Shrinkage

**Figure 10:**
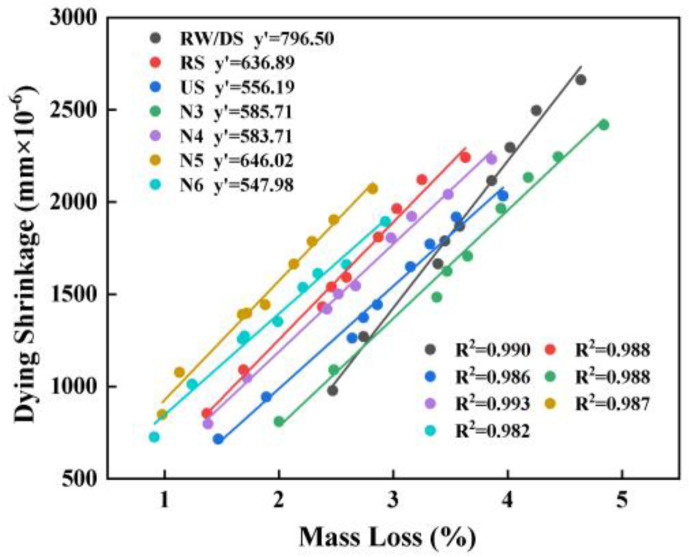
Fitted curve of mass loss vs. drying shrinkage.

Figure 10 demonstrates a strong positive correlation between drying shrinkage and mass loss, with an R^2^ value exceeding 0.98. This suggests that as the mass loss increases, the drying shrinkage also rises, driven by moisture dissipation and resulting capillary pressure within the mortar. The pore structure and moisture movement, influenced by factors such as the water and sand types and alkali equivalent, significantly affect the shrinkage. For instance, a higher alkali equivalent can lead to more hydration products, filling pores and impacting moisture loss, thereby affecting drying shrinkage [73]. These findings underscore the importance of optimizing the pore structure and hydration product formation to control drying shrinkage, enhancing the material’s stability and durability.

### 3.5. XRD

#### 3.5.1. Impact of Seawater and Sea Sand

**Figure 11:**
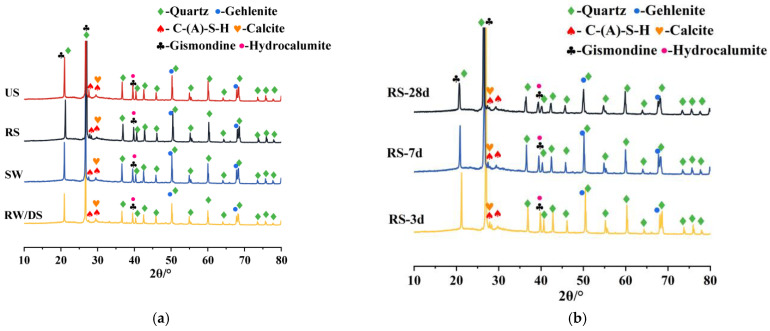

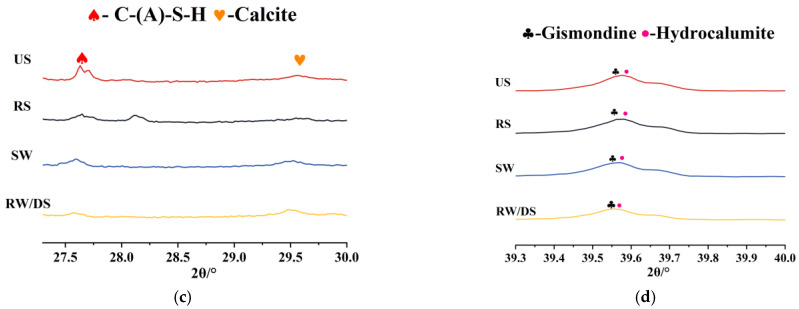
X-ray diffraction patterns of SSAS mortar: (**a**) groups with different water and sand types; (**b**) RS group at different ages; (**c**) enlarged peaks of C-(A)-S-H and calcite; (**d**) enlarged peaks of gismondine and hydrocalumite.

Figure 11a compares the XRD patterns of SSAS mortars prepared with different types of water and sand, highlighting the key components and hydration products. Quartz, a major component of sea sand, shows prominent diffraction peaks at 21°, 26.8°, 36.6°, and 50.1°, indicating the presence of SiO2. The main hydration products, C-S-H and C-(A)-S-H gels, exhibit broad peaks over 2θ = 15–40°, typical for SSAS mortar, with no significant differences among samples using different water sources [74].

Gehlenite (Ca_2_Al_2_SiO_7_) and gismondine (CaAl_2_Si_2_O_8_•4H_2_O), originating from the inert components and hydration reaction products of blast furnace slag, respectively, have their diffraction peaks located at 2θ = 50° and 70°; and 21°, 33.1°, and 39.5°, respectively. The presence of calcite (CaCO_3_) at 2θ = 30°, possibly caused by shell components in desalinated sea sand or carbonation reactions, affects the shrinkage properties of the mortar [75]. The formation of hydrocalumite (Ca_4_Al_2_O_6_Cl_2_•10H_2_O) is due to Cl^−^ replacing CO_3_^2−^ in the hydrocalcite. This substitution may further promote the formation of CaCO_3_ [76].

Mortar prepared with SW shows stronger diffraction peaks of gismondine and gehlenite, along with more pronounced C-S-H peaks, indicating that seawater promotes a more active hydration reaction, forming more hydration products, which play a key role in the samples’ excellent mechanical properties and lower drying shrinkage [77]. Due to its good crystallinity, an increase in gismondine crystals also acts to inhibit shrinkage. Furthermore, the higher chloride ion content in seawater leads to enhanced hydrocalumite peaks, which is beneficial in shrinkage suppression [72].

Regarding the different sand types, hydrocalumite peaks are absent in the RS group, which typically inhibit shrinkage. The remaining diffraction peaks are consistent across the groups, although their intensity and quantity vary. The RS group shows more C-S-H gel, suggesting better hydration and structural compactness. Prominent gismondine peaks at 2θ = 21° and 2θ = 39.2° in the US group explain its superior resistance to drying shrinkage. The reduction in shrinkage may also relate to the presence of calcite (CaCO_3_), which as a less reactive filler than slag can effectively reduce drying shrinkage [33]. However, due to the overlap between calcite and C-S-H gel peaks near 2θ = 30°, quantifying the calcite content among sand types is challenging. The TG analysis results in Section 3.6 provide further insights.

Figure 11b shows the XRD patterns of alkali-activated slag mortar with river sand at 3, 7, and 28 days. No new diffraction peaks appear across these ages in the RS group, indicating consistent hydration products. However, the intensity of the peaks for key hydration products, such as C-S-H, gehlenite, and gismondine, increases with age, particularly between 3 and 7 days. This suggests that hydration reactions and strength development in alkali slag mortar are most active during the early stages.

Furthermore, the formation of hydrocalumite through chloride binding, while beneficial for shrinkage resistance in non-reinforced applications, could pose risks in reinforced concrete due to potential chloride release. This further indicates SSAS’s particular suitability for non-reinforced applications such as concrete blocks.

#### 3.5.2. Impact of Alkali Equivalent

**Figure 12:**
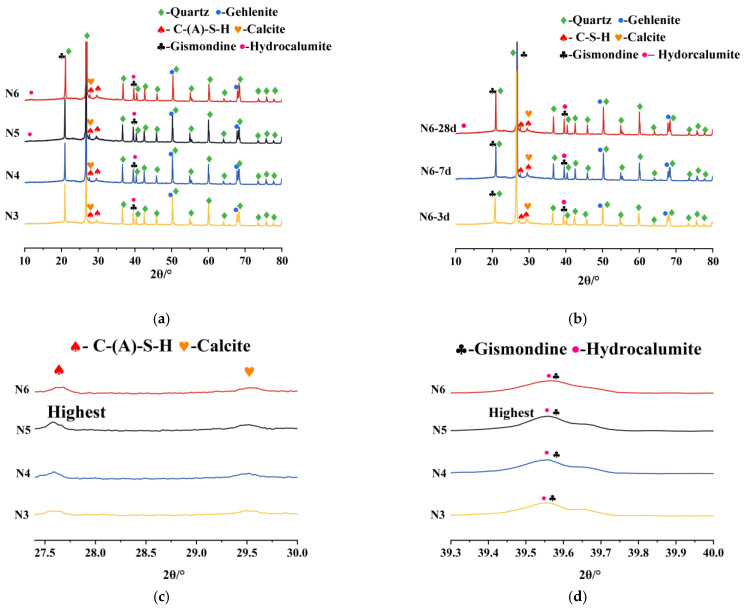
X-ray diffraction patterns of SSAS mortar: (**a**) groups with different alkali equivalents; (**b**) N6 group at different ages; (**c**) enlarged peaks of C-(A)-S-H and calcite; (**d**) enlarged peaks of gismondine and hydrocalumite.

To better understand how the alkali equivalent affects the SSAS mortar’s drying shrinkage, XRD patterns of samples with varying alkali equivalents at 28 days (Figure 12a) were analyzed. In the N6 and N5 groups, with higher alkali equivalents, hydrocalumite diffraction peaks around 2θ = 11° were observed. Hydrocalumite, also known as Friedel’s salt, plays a crucial role in trapping chloride ions in cementitious systems [78,79]. Its presence indicates an increased ability to bind chloride ions with higher alkali equivalents, leading to more chloride formation [80].

As the alkali equivalent increases, the peaks of C-(A)-S-H hydration products become more pronounced, suggesting improved slag hydration and the formation of additional hydration products. These products help fill voids in the mortar, promoting strength gain. The refinement of the pore structure due to the collapse and reorganization of C-(A)-S-H gel during drying enhances the mortar’s resistance to drying shrinkage [81]. Additionally, the stronger diffraction peaks of gismondine indicate improved crystallinity and stability, contributing to reduced shrinkage.

Figure 12b presents XRD patterns of the seawater–sea alkali-activated slag mortar with a 6% alkali equivalent at various ages. The N6 group’s hydration mainly occurs early, with later stages showing slower development. The overlap of hydrocalumite and gismondine peaks at 2θ = 39.5° complicates direct crystal quantity assessments. However, a small hump near 2θ = 11° at 28 days could hint at Friedel’s salt formation, indicating strengthening of the chloride-ion-binding capacity over time.

### 3.6. TG

#### 3.6.1. Impact of Seawater and Sea Sand

**Figure 13:**
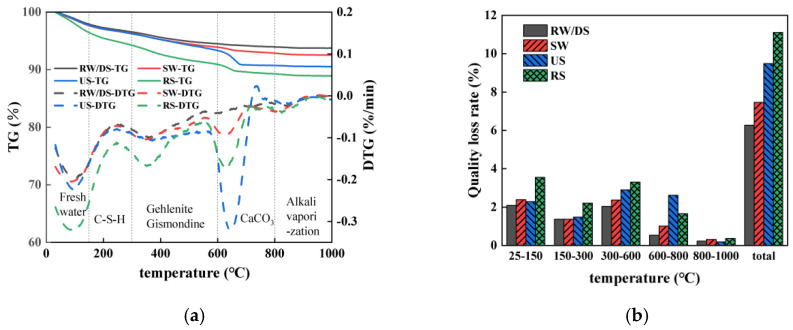
A TG analysis of SSAS mortar at 28 days under varying water and sand types: (**a**) TG-DTG images; (**b**) rate of mass loss.

Figure 13a displays the TG-DTG curves of SSAS mortars prepared with different sand types at 28 days. The process of mass loss is categorized into five stages: below 150 °C, 150–300 °C, 300–600 °C, 600–800 °C, and above 800 °C.

The first peak around 100 °C indicates free water loss [82,83,84], while the second stage (150–300 °C) shows mass loss due to the loss of chemically bound water in C-S-H [85,86], with a significant peak at 360 °C. The mass loss observed between 300 and 600 °C could be attributed to the anionic loss from hydration products such as gismondine [87], with a significant peak also emerging at 360 °C. In the 600–800 °C range, the mass loss corresponds to the decomposition of carbonate compounds [88]. This peak is more pronounced in the samples prepared with SW than with RW, suggesting that hydrocalumite formation in SW increases CO_3_^2−^ binding with Ca^2+^, leading to a distinct CaCO_3_ decomposition peak. The mass loss above 800 °C is likely due to the evaporation of the alkali solution [82].

Figure 13b shows the mass loss rates of each group across various temperature ranges. The SSAS mortars prepared using SW demonstrated higher mass loss rates compared to those prepared with RW, with the SW group exhibiting an 18.98% increase in total mass loss. In the 150–600 °C range, the higher mass loss in the SW group suggests a more intense hydration reaction, resulting in additional product formation that fills internal pores, creating a denser structure and inhibiting water transport [62]. Consequently, this also leads to enhanced compressive strengths in the specimens.

For different sand types, the mass loss rates in the 150–600 °C range rank as DS < US < RS, indicating that the RS group has more hydration products and a more compact structure, leading to lower drying shrinkage than in the DS group. In the 600–800 °C range, the US group shows the most significant mass loss due to CaCO_3_ decomposition, a major component of sea sand shells, contributing to the lowest drying shrinkage rate among the three groups [33].

Using a TG analysis, Ferone [89] identified the water-related components as free water below 150 °C and zeolitic water and hydroxyl water above 150 °C. The zeolitic and hydroxyl water are considered non-evaporative. Therefore, the evaporable water content for various mix proportions is determined using Formula (3). Here, *W*%_150–1000_ denotes the mass loss rate over the temperature range of 150–1000 °C. *W*%_tot_, on the other hand, refers to the total initial water content, which is calculated based on the amount of water incorporated during the preparation of the samples. The evaporable water content calculated for each test group is presented in Table 7.

(3) $$W=100-W{\%}_{150-1000}/W{\%}_{\mathrm{tot}}$$

A higher mass loss indicates more gel-bound water and less free water. The RW samples exhibited an 11.86% increase in evaporable water content compared to the SW samples, likely due to the slower hydration reaction in RW, resulting in less water being bound in the gel. This difference is also evident in the XRD patterns. A higher evaporable water content suggests more free water, leading to greater water loss under drying conditions and impacting RW’s drying shrinkage performance.

Among the different sand types, the evaporable water contents rank as DS > RS > US. The DS samples have the highest evaporable water content, with most water existing as interlayer and pore water rather than being bound to hydration products. This results in greater water loss and increased drying shrinkage in the DS samples. The comparative analysis shows a strong correlation between the evaporable water content and drying shrinkage in seawater–sea alkali slag mortars prepared with different sand types.

#### 3.6.2. Impact of Alkali Equivalent

**Figure 14:**
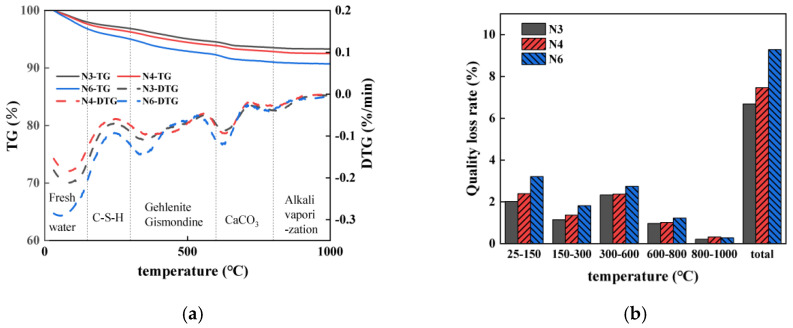
A TG analysis of SSAS mortar at 28 days under different alkali equivalents: (**a**) TG-DTG images; (**b**) rate of mass loss.

Figure 14 show the mass losses of mortars with alkali equivalents of 3%, 4%, and 6% across the temperature range of 25–1000 °C. The total mass loss increases with higher alkali equivalents, with an 11.68% increase when the alkali equivalent is raised from 3% to 4%, and a further 24.40% increase from 4% to 6%. For mortars with 4% and 6% alkali equivalents, the highest mass loss occurs between 25 and 150 °C, mainly due to the evaporation of free water. As the alkali equivalent rises, the rate of mass loss within this range increases, indicating a more significant loss of internal free water. In the 150–300 °C range, the mass loss also rises with higher alkali equivalents, reflecting more intense dehydration of C-S-H gel hydration products. Similarly, the mass loss intensifies in the 300–600 °C range, indicating the formation of more hydration products, which fill internal pores and reduce drying shrinkage. This densification also enhances the compressive strength in samples with higher alkali contents. However, there is a belief among some scholars that the formation of C-A-S-H gel can lead to increased shrinkage in concrete [90]. The specific impact of this gel on shrinkage properties is complex and requires further investigation, particularly in relation to porosity, which will be discussed later. Mass loss in the 600–800 °C range is due to decarbonation, with higher alkali equivalents leading to greater CaCO_3_ formation, as evidenced by the mass loss peak around 632 °C in all sample groups. The formation of CaCO_3_ may help mitigate shrinkage in groups with higher alkali equivalents [91].

To explore the relationship between drying shrinkage and the free water content in seawater–sea alkali slag mortar, the evaporable water content was calculated, as shown in Table 8. The data indicate a continuous decrease in evaporable water content as the alkali equivalent increases. Specifically, increasing the alkali equivalent from 3% to 4% results in a 5.16% reduction in evaporable water, and further increasing it from 4% to 6% leads to an additional 13.44% decrease. Higher alkali equivalents promote the formation of more hydration products, such as C-S-H gel, which increases the crystalline water content and reduces the free water content. This leads to a lower fraction of evaporable water, and consequently reduced drying shrinkage in drying environments. Specimens with higher alkali equivalents are less prone to water loss [62], which contributes to their decreased drying shrinkage.

#### 3.6.3. Relationship Between Evaporable Water Content and Drying Shrinkage

**Figure 15:**
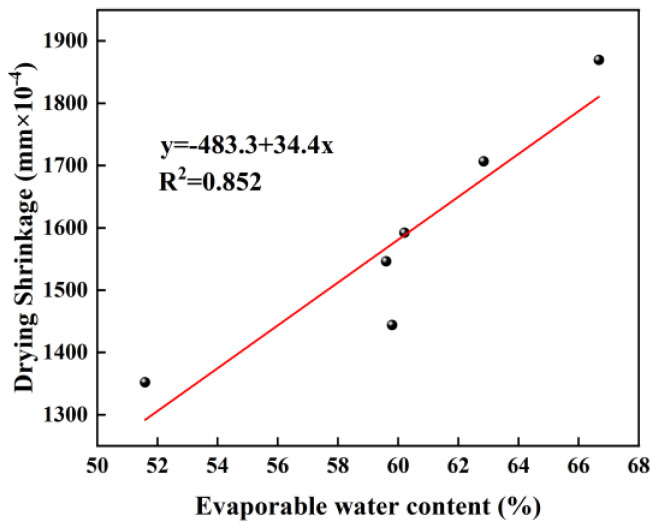
Fitting curve of evaporable water content vs. drying shrinkage.

Figure 15 presents a fitted graph of drying shrinkage versus the evaporable water content, with a correlation coefficient (R^2^) of 0.82, indicating a strong correlation. The fitted line has a slope of 34.4 and an intercept of −483.3, demonstrating a positive relationship between the evaporable water content and drying shrinkage. A higher evaporable water content in the samples suggests a reduced amount of gel-bound water during the hydration process, which translates to a greater proportion of free water. As a result, during the drying process, these samples are more susceptible to moisture loss, leading to increased drying shrinkage.

### 3.7. MIP

#### 3.7.1. Impact of Seawater and Sea Sand

**Figure 16:**
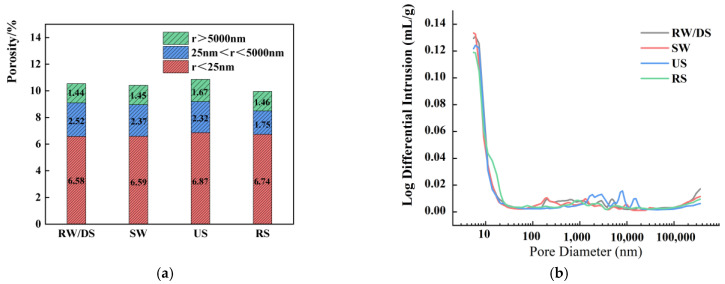
A pore structure analysis of SSAS samples with different water and sand types: (**a**) pore size distribution; (**b**) pore size differential diagram.

Figure 16 compare the pore size distribution and differential pore size images of SSAS mortars prepared with different types of water and sand. Compared to RW, the SW group shows lower overall porosity due to chloride ions accelerating the hydration reaction, filling pores with hydration products. The mesopore and microcrack porosity values of the SW group are nearly identical to the RW group but the SW group has fewer macropores, resulting in a finer pore size distribution and higher compressive strength [92,93].

For the different types of sand, the total porosity rates are ranked from low to high as RS < DS < US, indicating that the river sand group has the highest density and the best mechanical performance. Collins [94] pointed out that a larger volume proportion of mesopores in alkali slag is the reason for its greater drying shrinkage. Among the three samples, the mesopore porosity rates are ranked as DS < RS < US, yet US shows the lowest drying shrinkage. The difference in mesopore porosity rates among the samples is within 0.30%, and the pore size differential graph shows that smaller pores are concentrated at d < 20 nm, with no significant differences in distribution. This suggests that the mesopore volume has a limited impact on the drying shrinkage of US, which is mainly influenced by changes in phase composition.

#### 3.7.2. Impact of Alkali Equivalent

**Figure 17:**
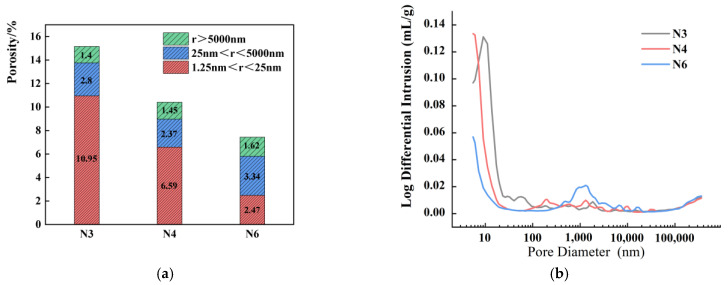
A pore structure analysis of SSAS samples with different alkali equivalents: (**a**) pore size distribution; (**b**) pore size differential diagram.

Figure 17 illustrate the 28-day pore size distribution characteristics of SSAS mortars with varying alkali equivalents. The images show that as the Na_2_O content increases, the overall porosity of the system decreases. Specifically, increasing the Na_2_O content from 3% to 4% reduces the total porosity by 31.24%, and further increasing it to 6% results in a 50.99% reduction. This suggests that a higher Na_2_O content refines the pore structure, as hydration products fill the pores, enhancing the compressive strength.

Regarding the mesoporous porosity, with 3% alkali equivalent, it measures 10.95%, while at 4% and 6%, it decreases to 6.59% and 2.47%, respectively. This indicates that higher alkali equivalents significantly impact the 28-day pore size distribution, with both the mesoporous and total porosity decreasing. Consequently, reduced mesoporous porosity at higher alkali equivalents lowers the capillary pressure, leading to reduced drying shrinkage [91].

With the increase in alkali equivalent, a significant reduction in the number of pores smaller than 400 nm can be observed in the high alkali equivalent N6 group, distinguishing it from the other groups. This observation implies that the influence of hydration products on pores is not restricted to refining larger pores; it also extends to reducing the porosity of mesopores. Additionally, the pore size distribution in the N4 and N5 groups is concentrated in the region where the diameter (d) is less than 20 nm, whereas the pores in the N6 group exhibit a more uniform distribution. As a result, this leads to reduced shrinkage and enhanced mechanical properties.

### 3.8. SEM

#### 3.8.1. Impact of Seawater and Sea Sand

**Figure 18:**
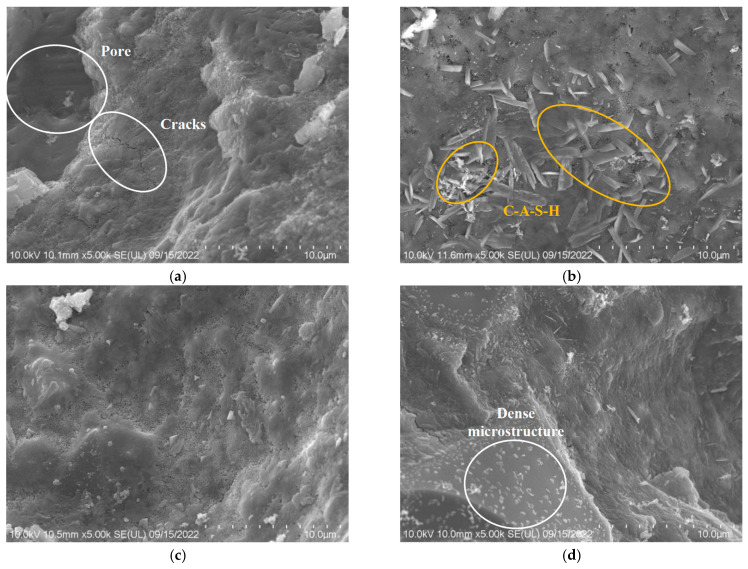
SEM images of SSAS mortars with different water and sand types: (**a**) RW/DS; (**b**) SW; (**c**) US; (**d**) RS.

Figure 18 display the microstructures of mortars prepared with different types of water. From the SEM images, we can observe various morphologies of hydration products. These morphologies include flaky, reticulated, prismatic, cubic, and other irregular forms of hydration products. In the samples prepared with seawater, there is a noticeable presence of C-A-S-H gels and some needle-like hydration products. The abundance of these hydration products contributes to the filling of pores, ultimately resulting in lower shrinkage values.

The microstructures of the alkali slag mortars prepared with different types of sand are depicted in Figure 18c,d. In Figure 18a, the use of desalinated sea sand results in the presence of some microcracks and unreacted slag particles. These unreacted slag particles can be distinguished by their sharp, irregular shapes and bright gray tones [95]. However, when undiluted sea sand is used, the SEM images reveal the disappearance of microcracks, and there are fewer unreacted slag particles, resulting in a more compact sample. Upon comparing Figure 18c,d, it becomes evident that the sample prepared with river sand exhibits the most compact microstructure, and the unreacted slag particles are smaller, adhering to the surface of the hydration products, as shown in Figure 18d. Consequently, the alkali slag mortar prepared with river sand demonstrates the highest mechanical performance among the three groups of samples. Both the DS and RS samples exhibit a minimal number of microcracks, which are attributed to the tensile stress generated by their higher drying shrinkage [96]. This lower drying shrinkage contributes to reduced overall shrinkage.

#### 3.8.2. Impact of Alkali Equivalent

**Figure 19:**
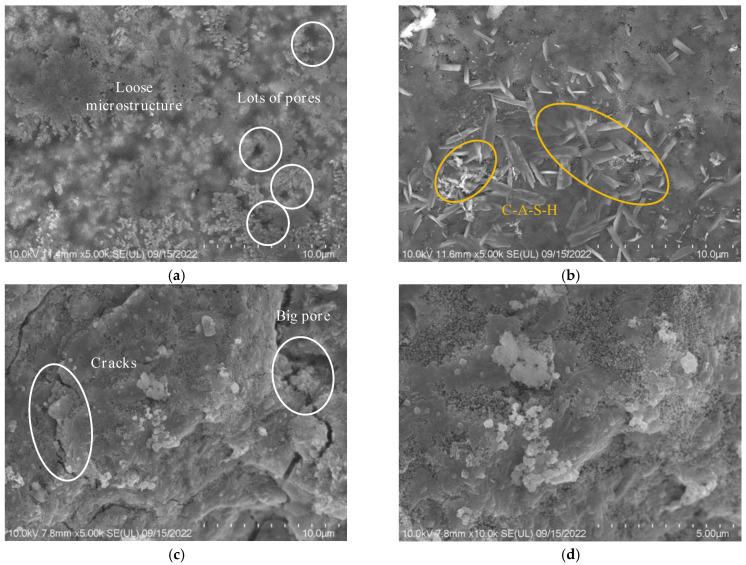
SEM images of SSAS mortars with different alkali equivalents: (**a**) N3; (**b**) N4; (**c**) N6; (**d**) N6 enlarged image.

Scanning electron microscope tests were conducted on sea water sea sand alkali slag mortars with alkali equivalents of 3%, 4%, and 5% to understand the differences in hydration products and micro-morphologies resulting from different alkali equivalents. Figure 19a reveals that groups with lower alkali equivalents exhibit fewer hydration products and the presence of some pores in the microstructure. On the other hand, Figure 19c indicates the presence of noticeable cracks in samples with an alkali equivalent of 6%. This could be attributed to the excessive alkali content, which negatively affects the workability during the sample preparation process, leading to an increased number of voids and the formation of cracks [92]. These cracks may be responsible for the reduced flexural strength observed at high alkali equivalents. Additionally, the excessive dissolution of sodium silicate particles in the environment can exert pressure on the matrix, resulting in the formation of a significant number of voids. The presence of these voids indicates the complete dissolution of sodium silicate [96]. A comparison of Figure 19a,c reveals that the density of the observed hydration product C-S-H gel, under the same magnification conditions, increases in the order of N3 < N4 < N6, with N6 exhibiting higher compressive strength.

## 4. Conclusions

This research examined the influence of the water type, sand type, and alkali equivalents on the macroscopic characteristics of seawater–sea sand alkali-activated slag mortar. This comprehensive investigation encompassed aspects such as the flowability, mechanical properties, drying shrinkage, and mass loss. Additionally, microscopic analyses were performed using techniques such as XRD, TG, MIP, and SEM to elucidate the underlying mechanisms. The primary findings are summarized as follows:

(1) The use of seawater enhances the mechanical properties of alkali-activated slag mortar mainly through the increased formation of hydration products, driven by the accelerating effect of chloride ions on hydration reactions. This leads to a denser material by filling the pores with hydration products, including C-A-S-H gel, which reduces porosity. The presence of gismondine contributes to shrinkage inhibition. Moreover, seawater reduces the content of evaporable water, minimizing moisture loss during drying and reducing drying shrinkage. This is entirely consistent with the research results of other scholars.

(2) While sea sand marginally affects the workability and leads to reduced strength, river sand offers superior mechanical performance due to its smoother surface, which enhances bonding. The incorporation of undesalinated sea sand leads to the lowest mechanical performance due to the presence of shell impurities; however, these contribute to reduced drying shrinkage by lowering the evaporable water content. However, due to differences in shell hardness and the presence of impurities, different trends may be observed in different studies.

(3) Using undesalinated sea sand in the mortar results in lower drying shrinkage. This effect is attributed to the lower evaporable water content and the mitigating role of shell impurities in undesalinated sea sand, which lead to fewer microcracks. Desalinated sea sand, with its fine particles, demands more water, leading to a higher evaporable water content and consequently increased drying shrinkage. This suggests that selecting the appropriate type of sea sand can address the issue of high drying shrinkage rates.

(4) Increasing the alkali equivalent from 3% to 6% enhances the compressive strength of the mortar but negatively affects the flexural strength over time. This increase in alkali content promotes more vigorous hydration reactions, resulting in denser hydration products and improved mechanical properties. Additionally, a higher alkali content leads to decreased evaporable water and mesopore porosity levels, achieving the lowest drying shrinkage with the highest alkali equivalents tested (6%). Other scholars believe that the optimal alkali equivalent rate may be related to different alkali modulus values, depending on the pore structure of the product. The findings highlight the importance of optimizing the alkali content to balance strength development and drying shrinkage control to achieve the best performance for alkali-activated slag mortar.

## Figures and Tables

**Table 1:** Physical properties of three types of sand.

Physical Property	DS	US	RS
moisture content/%	0.28	0.54	4.80
mud content/%	1.20	1.52	2.46
Chloride ion content/%	0.050	0.163	0.003
Shell content/%	1.76	11.20	/
Fineness modulus	1.84	2.83	3.03
Apparent density/kg/m^3^	2650	2640	2670

**Table 2:** Chemical composition of sand (g/L).

Chemical Composition%	SiO_2_	CaO	Al_2_O_3_	K_2_O	Fe_2_O_3_	MgO	Na_2_O
DS	90.34	3.52	2.02	1.00	0.48	0.36	0.18
US	87.41	4.64	1.98	1.02	0.51	0.34	0.25
RS	93.70	2.16	2.22	0.93	0.44	0.53	0.12

**Table 3:** Chemical composition of seawater (g/L).

NaCl	MgCl_2_	Na_2_SO_4_	CaCl_2_	KCl	NaHCO_3_
24.53	5.2	4.09	1.16	0.695	0.201

**Table 4:** Basic physical quantities of slag.

Physical Quantities	Densities (g/cm^3^)	Specific Surface Area (m^2^/kg)	7 Days Activity Index (%)	28 Days Activity Index (%)	Heat Loss (%)	Chloride Ion Content (%)	Mobility Ratio (%)	Moisture Content (%)
Data	2.92	425.00	78.00	96.00	1.02	0.03	103.00	0.36

**Table 5:** Chemical composition of slag/%.

Oxide (%)	CaO	SiO_2_	Al_2_O_3_	MgO	SO_3_	TiO_2_	Na_2_O	Fe_2_O_3_	MnO	K_2_O	Cl	SrO	Others
Slag	36.837	30.266	17.18	9.67	2.789	1.249	0.596	0.483	0.402	0.336	0.051	0.039	0.102

**Table 6:** SSAS mortar ratio.

Number	Group Name	Type of Water	Type of Sand	Water–Binder Ratio	Type of Alkali	Alkali Equivalent
1	RW	RW	DS	0.43	Na_2_SiO_3_	4%
2	SW	SW	DS	0.43	Na_2_SiO_3_	4%
3	DS	RW	DS	0.43	Na_2_SiO_3_	4%
4	US	RW	US	0.43	Na_2_SiO_3_	4%
5	RS	RW	RS	0.43	Na_2_SiO_3_	4%
6	N3	SW	DS	0.43	Na_2_SiO_3_	3%
7	N4	SW	DS	0.43	Na_2_SiO_3_	4%
8	N5	SW	DS	0.43	Na_2_SiO_3_	5%
9	N6	SW	DS	0.43	Na_2_SiO_3_	6%

**Table 7:** Effect of seawater on evaporable water content of SSAS mortar.

Groups	RW/DS	SW	US	RS
Evaporable water content/%	66.67	59.60	59.80	60.21

**Table 8:** Effect of alkali equivalent on evaporable water content of SSAS mortar.

Groups	N3	N4	N6
Evaporable water content/%	62.84	59.60	51.59

## Data Availability

The original contributions presented in this study are included in the article. Further inquiries can be directed to the corresponding author(s).
